# Editorial: *In vivo* Cell Biology of Cerebral Cortical Development and Its Related Neurological Disorders

**DOI:** 10.3389/fncel.2016.00162

**Published:** 2016-06-21

**Authors:** Takeshi Kawauchi, Margareta Nikolić, Yoko Arai

**Affiliations:** ^1^Laboratory of Molecular Life Science, Institute of Biomedical Research and Innovation, Foundation for Biomedical Research and InnovationKobe, Japan; ^2^Department of Physiology, Keio University School of MedicineTokyo, Japan; ^3^Precursory Research for Embryonic Science and Technology, Japan Science and Technology AgencySaitama, Japan; ^4^Department of Biological and Environmental Sciences, School of Life and Medical Sciences, University of HertfordshireHatfield, UK; ^5^PROTECT, INSERM, Unversité Paris Diderot, Sorbonne Paris CitéParis, France; ^6^Institut Jacques-Monod, Centre National de la Recherche Scientifique UMR 7529, Université Paris DiderotParis, France

**Keywords:** neurogenesis, neuronal migration, cell division, cytoskeleton, endocytosis, microcephaly, periventricular heterotopia, lissencephaly

The brain consists of complex but precisely organized neural networks, which determine the structural basis of higher order functions. Remarkably, this complex structure originates from a simple pseudostratified neuroepithelium. How it is formed is best seen in the elegant example of the cerebral cortex. In the developing mammalian cerebral cortex, polarized neural progenitors are arranged in a pseudostratified structure that forms the mitotically active ventricular zone. At the onset of neurogenesis, a cohort of neural progenitors differentiates into neurons and through multi-step modes of migration generates a six-layered structured cerebral cortex. Defects in neurogenesis and neuronal migration can cause several neurological disorders, including microcephaly and lissencephaly. Importantly, recent advances in not only human and mouse genetic approaches but also the use of a number of novel techniques, particularly *in vivo* electroporation and time-lapse analyses of explant slice culture, have significantly increased our understanding of cortical development. In addition, these novel techniques have allowed us to open a new avenue for cell biological analyses of cortical development *in vivo* or *ex vivo*.

The aim of this research topic is to highlight important mechanisms underlying cerebral cortical development and associated neurological disorders, with a specific focus on cell biology, including cell division, cell cycle regulation, cytoskeletal organization, cell adhesion, endocytosis, and membrane trafficking. The topic has been organized into three sections: (1) neurogenesis and cell fate determination, (2) neuronal migration, and (3) cortical development-related neurological disorders.

The first section highlights cellular insights into neurogenesis and cell fate determination. In the developing cerebral cortex, apical neural progenitors (radial glial progenitors) exhibit cell cycle-dependent nuclear movement, termed interkinetic nuclear migration (INM). Rho family small GTPases, including Rac1 and Rnd3, are known to control the proliferation and INM of apical progenitors (Azzarelli et al). While a physiological significance of INM remains unclear, it is thought that INM is associated with the nuclear traffic control in the ventricular zone (Miyata et al).

At M phase, apical progenitors undergo proliferative symmetric or neurogenic asymmetric division along the ventricle. The precise control of the balance between symmetric and asymmetric cell division is required for the proper production of neurons during cortical development. Recently, spindle size asymmetry has been suggested as a core component to regulate the asymmetric cell division (Delaunay et al). Interestingly, spindle size asymmetry in neural progenitors is observed both in mouse and macaque cerebral cortices, suggesting a conserved, and thus important mechanism during mammalian cortical evolution. Another feature of asymmetric cell division is a release of the midbody, a cytoplasmic bridge between two daughter cells at the end of mitosis. While midbody retention is for the maintenance of proliferative capacity of the cells, the midbody release is associated with the progression of differentiation/neurogenesis. Proliferative cancer cells retain the midbody carrying a phospholipid, phosphatidylserine (PS), geometrically inside of the plasma membrane, however, differentiating cells and apical progenitors release the midbody carrying PS outside of the plasma membrane (Arai et al). This PS asymmetry in midbody allowed cells to take midbody up by themselves or loose them to regulate their differentiation process.

Several transcription factors are involved in controlling the balance between proliferation and differentiation of neural progenitors. Among them, Pax6 is highly expressed in apical progenitors in the ventricular zone of the developing cerebral cortex and regulates their proliferation and cell-cycle exit to generate cortical excitatory projection neurons (Manuel et al.). In the ganglionic eminence and preoptic area (POA), Nkx2.1 and Nkx5.1 transcription factors play pivotal roles in the production of cortical inhibitory interneurons (Peyre et al.). Recent reports show that a temporal oscillatory nature of basic helix-loop-helix (bHLH) transcription factors, such as Ascl1/Mash1, Hes1, Neurogenin2, and Olig2, play important roles in fate determination. Apical progenitors co-express several bHLH transcription factors in an oscillatory expression pattern. Upon the determination of cell fate, one of bHLH transcription factors dominates and is continuously expressed (Imayoshi et al.).

Apical progenitors give rise to either neuronally-committed intermediate progenitor cells or immature cortical excitatory neurons by losing their cell polarity. An Axin-GSK3β complex controls the generation and proliferation of intermediate progenitor cells (Ye et al). Interestingly, the delamination from the pseudostratified neuroepithelium or ventricular zone resembles an epithelial–mesenchymal transition (EMT) and requires the down-regulation of several cell adhesion molecules, such as cadherins, nectins, and junctional adhesion molecules (JAMs) (Singh and Solecki).

The second section highlights cellular insights into the migration of excitatory neurons and inhibitory interneurons from the dorsal ventricular zone or ventral ganglionic eminence, respectively. Newly generated excitatory neurons from the dorsal ventricular zone first display a multipolar morphology, which requires Cx43, a gap junction protein, and p27 that acts as a cytoskeletal regulator rather than a cell cycle inhibitor (Cooper). Subsequently, neurons transform into a bipolar form by extending an axon and a pia-directed leading process and retracting all other processes. Many molecules regulating the axon formation during the multipolar-to-bipolar transition have been identified (Cooper). LKB1 and its associated molecules, Stk25 and STRADα, are reported to regulate axon outgrowth. Interestingly, under the control of an atypical cyclin-dependent kinase (Cdk5), Axin, and GSK3β also play a role in axon formation (Ye et al.).

The bipolar neurons, called locomoting neurons, migrate along apical progenitor-derived long radial fibers with unique morphological changes (Kawauchi). The attachment of neurons to the radial fibers and the neuron-specific migration mode require N-cadherin-mediated adhesion and the Cdk5–Dcx/p27 pathway, respectively. At the final phase of neuronal migration, neurons change their migration mode into a radial fiber-independent terminal translocation mode, which is controlled by a secreted molecule, Reelin, and its downstream cytoplasmic adaptor, Dab1 (Yap and Winckler).

In contrast to the excitatory projection neurons, immature inhibitory interneurons, born at the ganglionic eminence or POA, migrate tangentially to the cerebral cortex (Luhmann et al.; Peyre et al.). Immature cortical inhibitory and excitatory neurons partly share migration mechanisms at least on a molecular level. For example, Cdk5, p27, Dcx, N-cadherin, and Rho family small GTPases are known to regulate both types of neuronal migration (Azzarelli et al.; Cooper; Kawauchi; Luccardini et al.; Peyre et al.; Ye et al.). However, inhibitory interneurons display branched leading processes and their tangential migration is not dependent on radial fibers, suggesting that specific mechanisms are also required for their movement. Interestingly, neurotransmitters, glutamate, and GABA, and their receptors are known to control neuronal migration (Luhmann et al.), and glycine α2 receptor is required for the migration of cortical inhibitory interneurons via the fine-tuning of acto-myosin contraction during nucleokinesis (Peyre et al.).

Neuronal migration depends on dynamic regulation of a huge number of intracellular and membrane proteins. Microtubule and actin cytoskeletal organization are essential for the morphological changes of migrating neurons and dysregulation of the cytoskeletons can result in several neurological disorders (Lian and Sheen, Peyre et al.). For example, axophilic migration of GnRH neurons requires cooperation of cortical actin flow and microtubule organization, defect in which causes Kallmann syndrome, a neuroendocrine disorder (Hutchins and Wray). In addition, recent reports have indicated that endocytosis and membrane trafficking pathways regulate several steps of neuronal migration (Kawauchi, Yap and Winckler). Endocytic pathway-mediated regulation of N-cadherin plays an essential role in the radial fiber-dependent migration of cortical excitatory neurons (Kawauchi). The tangential migration of cortical inhibitory interneurons, which is independent of radial fibers, also requires N-cadherin (Luccardini et al.). Thus, the multi-step neuronal migration and morphological changes rely on coordinated regulation of various cellular events.

The third section highlights cellular insights into cortical development-related neurological disorders. Disruption of the proper balance between proliferation and differentiation results in cortical malformations, such as microcephaly or megalencephaly (Bizzotto and Francis). Increased cell death of neural progenitors can also lead to microcephaly. At least 12 causative genes have been linked to autosomal recessive primary microcephaly. The loci are numbered by *MCPH1*–*MCPH12*. MCPH1 controls the centrosome cycle through the Chk1–Cdc25 pathway and DNA damage repair, whose defects may lead to the development of a small brain (Pulvers et al.). Mutations of *ASPM* (*MCPH5*) are the most common cause of autosomal recessive primary microcephaly in humans, and ASPM protein shows important roles in maintaining the spindle positioning during the mitosis of neural progenitors (Bizzotto and Francis, Pulvers et al.).

Abnormalities in apical progenitors can also result in cobblestone (type II) lissencephaly or periventricular heterotopia, caused by defects in the attachment of basal processes (radial fibers) to the pial surface or from disruptions in the apical (ventricular) surface, respectively. Globular heterotopia, which was observed in *Eml1* homozygous mutant or *N-cadherin Emx1*^*Cre*^ conditional knockout mice, occurs when apical progenitors detach from the ventricular surface (Bizzotto and Francis).

*Filamin A* and *ArfGEF2*, whose gene products regulate actin cytoskeleton and membrane trafficking, respectively, are reported as causative genes for periventricular heterotopia (Lian and Sheen). Mutations in genes encoding microtubule-regulatory proteins, such as *Lis1* and *Dcx*, result in type I lissencephaly (Kawauchi). In addition to these cell intrinsic factors, prenatal environmental stresses, such as alcohol, hypoxia, and exposure to heavy metals, can induce cortical malformation and the impairment of cognitive and memory functions (Ishii and Hashimoto-Torii). Interestingly, these types of environmental stress activate intracellular stress response signaling, including the heat shock protein (HSP)-mediated pathway. Thus, dysregulation of various cellular events is closely associated with neurological disorders.

One of the strategies for overcoming these neurogenesis- or neuronal migration-related neurological disorders is to re-activate neurogenesis in the postnatal cerebral cortex. Despite the obvious challenges, reprogramming of astrocytes (or neurons) into specific neuronal subtypes may be one important approach for brain repair (Akhtar and Breunig).

This research topic aims to provide multidisciplinary approaches, encompassing developmental neuroscience and cell biology to understand mechanisms of cortical development as well as an etiology of neurological and psychiatric disorders. We hope that results and knowledge provided by all authors in this research topic will be useful for patients' care, as well as future advances in basic research.

## Author contributions

TK, MN, and YA wrote the manuscript.

### Conflict of interest statement

The authors declare that the research was conducted in the absence of any commercial or financial relationships that could be construed as a potential conflict of interest.

